# New drugs targeting Th2 lymphocytes in asthma

**DOI:** 10.1186/1745-6673-3-S1-S6

**Published:** 2008-02-27

**Authors:** Gaetano Caramori, David Groneberg, Kazuhiro Ito, Paolo Casolari, Ian M Adcock, Alberto Papi

**Affiliations:** 1 Dipartimento di Medicina Clinica e Sperimentale, Centro di Ricerca su Asma e BPCO, Università di Ferrara, Ferrara, Italy; 2 Institute of Occupational Medicine, Charité- Universitätsmedizin Berlin, Free University and Humboldt University, Berlin, Germany; 3 Airway Disease Section, National Heart and Lung Institute, Imperial College of London, London, UK

## Abstract

Asthma represents a profound worldwide public health problem. The most effective anti-asthmatic drugs currently available include inhaled β2-agonists and glucocorticoids and control asthma in about 90-95% of patients. The current asthma therapies are not cures and symptoms return soon after treatment is stopped even after long term therapy. Although glucocorticoids are highly effective in controlling the inflammatory process in asthma, they appear to have little effect on the lower airway remodelling processes that appear to play a role in the pathophysiology of asthma at currently prescribed doses. The development of novel drugs may allow resolution of these changes. In addition, severe glucocorticoid-dependent and resistant asthma presents a great clinical burden and reducing the side-effects of glucocorticoids using novel steroid-sparing agents is needed. Furthermore, the mechanisms involved in the persistence of inflammation are poorly understood and the reasons why some patients have severe life threatening asthma and others have very mild disease are still unknown. Drug development for asthma has been directed at improving currently available drugs and findings new compounds that usually target the Th2-driven airway inflammatory response. Considering the apparently central role of T lymphocytes in the pathogenesis of asthma, drugs targeting disease-inducing Th2 cells are promising therapeutic strategies. However, although animal models of asthma suggest that this is feasible, the translation of these types of studies for the treatment of human asthma remains poor due to the limitations of the models currently used. The myriad of new compounds that are in development directed to modulate Th2 cells recruitment and/or activation will clarify in the near future the relative importance of these cells and their mediators in the complex interactions with the other pro-inflammatory/anti-inflammatory cells and mediators responsible of the different asthmatic phenotypes. Some of these new Th2-oriented strategies may in the future not only control symptoms and modify the natural course of asthma, but also potentially prevent or cure the disease.

## Introduction

Asthma represents a profound worldwide public health problem. The most effective anti-asthmatic drugs currently available include inhaled β2-agonists and glucocorticoids and control asthma in about 90-95% of patients. However, the future therapies will need to focus on the 5-10% patients who do not respond well to these treatments and who account for ∼50% of the health care costs of asthma [1,2]. Strategies for the primary prevention of asthma remain in the realm of speculation and hypothesis [3]. Drug development for asthma has been directed at improving currently available drugs and findings new compounds that usually target the Th2-driven airway inflammatory response. Several new compounds have been developed to target specific components of the inflammatory process in asthma [e.g. anti-IgE antibodies(omalizumab), cytokines and/or chemokines antagonists, immunomodulators, antagonists of adhesion molecules)], although they have not yet been proven to be particularly effective. In fact only omalizumab has reached the market where it may be most cost-effective in those patients with severe persistent asthma and frequent severe exacerbations requiring hospital care [3-5]. In this chapter we will review the role of current antiasthma drugs and future new chemical entities able to target Th2 cells in asthmatic airways. Some of these new Th2-oriented strategies may in the future not only control symptoms and modify the natural course of asthma, but also potentially prevent or cure the disease.

## Effects of current antiasthma drugs on Th2 cells in asthmatic airways

Despite the large number of controlled clinical studies on the effect of many antiasthma drugs (particularly inhaled glucocorticoids) in suppressing airway inflammation in asthmatics, there is a complete absence of controlled clinical studies on the effect of these drugs on the Th2/Tc2 lymphocytes ratio in the airways of asthmatic patients. In particular it is still unknown if inhaled glucocorticoids can decrease the recruitment of Th2 lymphocytes and/or the degree of their differentiation and/or activation.

*In vivo* animal models of asthma, particularly murine, have been increasingly used to investigate the efficacy of several anti-asthma drugs, including their effect on Th2 lymphocytes. However, animal models of asthma have limitations; most are models of acute allergen exposure which are sensitive to anti-interleukin(IL)-5 strategies; animals do not spontaneously develop asthma and no model mimics the entire asthma phenotype [6]. For these reasons, the results obtained in animal models of asthma must be confirmed with controlled clinical trials in asthmatic patients.

## Effects of glucocorticoids on Th2 cells

Inhaled glucocorticoids are the only anti-asthma agents that clearly reverse the specific chronic airway inflammation present in asthma. Inhaled glucocorticoids have anti-inflammatory effects in the airway of patients with asthma [3]. In patients treated with inhaled glucocorticoids there is a marked reduction in the number of mast cells, macrophages, T-lymphocytes, and eosinophils in the sputum, bronchoalveolar lavage (BAL) and bronchial wall [7,8]. Furthermore, glucocorticoids reverse the shedding of epithelial cells, the mucus-cell hyperplasia and basement-membrane thickening characteristically seen in biopsy specimens from patients with asthma [7,8]. However some inflammation still persists in the airways of patients with asthma who have poor airway function, despite regular and prolonged treatment with high doses of inhaled or systemic glucocorticoids [8,9]. The inflammatory component of asthmatic airways most responsive to glucocorticoid treatment seems to be eosinophilic inflammation. In patients with persistent asthma, well controlled tapering of inhaled glucocorticoids induces an exacerbation within a few months. This is usually associated with a reversible increase of eosinophilic airway inflammation. Some patients with difficult-to-control asthma may develop exacerbations despite treatment with inhaled glucocorticoids, and these often appear to have an eosinophil-independent inflammatory mechanism [8,9].

Glucocorticoids also have direct inhibitory effects on many of the cells involved in airway inflammation in asthma, including macrophages, T-lymphocytes, eosinophils, mast cells, and airway smooth muscle and epithelial cells. *In vitro*, glucocorticoids decrease cytokine mediated survival of eosinophils by stimulating apoptosis.

This process may explain the reduction in the number of eosinophils, particularly low density eosinophils, in the circulation and lower airways of patients with asthma during glucocorticoid therapy. Inhaled glucocorticoids attenuated the allergen-induced increase in peripheral blood eosinophils and on eosinophil/basophil colony-forming units (Eo/B CFU) [7,8]. They also significantly attenuated the baseline, but not allergen-induced increase, numbers of total CD34(+) cells, CD34(+)IL-3Rα+ cells and interleukin (IL)-5-responsive Eo/B-CFU in the bone marrow. Glucocorticoids may not inhibit the release of mediators from mast cells, but they do reduce the number of mast cells within the airway [7,8]. CD4+ and CD8+ T lymphocytes in peripheral blood of asthmatic patients are in an activated state and this is down regulated by inhaled glucocorticoids. In fact, treatment with inhaled glucocorticoids reduces the expression of the activation markers CD25 and HLA-DR in both CD4+ and CD8+ T-cell subsets in peripheral blood of patients with asthma. In addition, there is correlation between the down regulation of CD4 and CD8 T-lymphocyte activation and the improvement in asthma control. Treatment with inhaled glucocorticoids reduces the number of activated T lymphocytes (CD25+ and HLA-DR+) in the BAL from asthmatic patients [7,8]. However, severe glucocorticoid-dependent and resistant asthma is associated with persistent airway T-lymphocyte activation [9-11].

In general, glucocorticoids substantially reduce the mast cell/eosinophil/lymphocyte driven processes, while leaving behind or even augmenting a neutrophil-mediated process. Glucocorticoids enhance neutrophil function through increased leukotriene and superoxide production, as well as inhibition of apoptosis. Glucocorticoids have no effect on sputum neutrophil numbers in patients with severe persistent asthma [9]. Part of the anti-inflammatory activity of glucocorticoids in asthma may also involve reduction in macrophage and resident cell eicosanoid (leukotriene B_4_ and thromboxane B_2_) and cytokine and chemokine (e.g. IL-1β, IL-4, IL-5, IL-8, GM-CSF, TNF-α, CCL3 [macrophage inflammatory protein-1alpha (MIP-1α)] and CCL5 (RANTES) synthesis [7,12]. In addition to their suppressive effects on inflammatory cells, glucocorticoids may also inhibit plasma exudation and mucus secretion in inflamed airways. However, glucocorticoids have no effect on sputum concentrations of fibrinogen. There is an increase in vascularity in the bronchial mucosa of asthmatics and high doses of inhaled glucocorticoids, may reduce airway wall vascularity in asthmatic patients. Inhaled glucocorticoids also attenuate the increased airway mucosal blood flow present in asthmatic patients [7,8].

Many *in vitro* studies have indicated that glucocorticoids may participate in guiding the differentiation of T lymphocytes toward the Th2 phenotype [13]. The immunosuppressive effect of glucocorticoids after organ transplantation is mainly due to preferential blockade of Th1 cytokine expression and promotion of a Th2 cytokine-secreting profile. Glucocorticoids, *in vitro* (a) inhibit IL-12 secretion from monocyte-macrophages and dendritic cells, (b) decrease IL-12 receptor 1- and 2-chain expression, thereby inhibiting IL-12 signaling, and (c) inhibit IL-12-induced STAT-4 (transcription factor that drives Th1 differentiation) phosphorylation without affecting STAT-6 (transcription factor that drives Th2 differentiation) phosphorylation (d), and thereby deviate the immune response predominantly toward the Th2 phenotype [8,12].

In stable asthmatics systemic glucocorticoid treatment produces a small but significant decrease of 16% in blood CD3+CD4+ and a 12% increase in natural killer(NK)-cell frequency within 3 hours. In contrast, the CD3+CD8+ T-cell number and activation marker remains unchanged [14]. *In vitro* fluticasone inhibits IL-5 and IL-13 and enhances IL-10 synthesis in allergen-stimulated peripheral blood CD4+ T cell cultures in a concentration-dependent manner [15]. Similarly, salmeterol, but not salbutamol, also inhibits IL-5 and IL-13 and enhances IL-10 synthesis in the same cultures [15]. When used in combination the two drugs demonstrated an additive effect on this pattern of cytokine production [15] perhaps through an effect on NFAT and AP-1 transcription factors [16].

Furthermore, *in vitro*, glucocorticoids inhibit proliferation and IL-4 and IL-5 secretion by human allergen-specific Th2 lymphocytes [17]. Both beclomethasone and fluticasone inhibit allergen-induced peripheral blood T-cell proliferation and their expression of IL-5 and GM-CSF in asthmatics [18].

Interestingly, the combination of fluticasone and salmeterol significantly inhibits production of IFN-γ, but not that of Th2 cytokines (IL-5 and IL-13) from PBMCs from asthmatic subjects [19]. This is in contrast with the results of an earlier study [20]. When rolipram, a phosphodiesterase 4 inhibitor, is added to the fluticasone-salmeterol combination, this triple combination inhibits IL-13 production by PBMCs from asthmatic patients [19].

*In vitro* fluticasone alone increases and salmeterol alone does not affect peripheral blood T-cell apoptosis in either normal or asthmatic subjects [21,22]. Their combination significantly increases peripheral blood T-cell apoptosis in comparison with fluticasone alone and it is also able to reduce the expression of the phosphorylated inhibitory κB alpha (IκBα), thus limiting nuclear factor κB (NF-κB) activation [22].

## Effects of theophylline on Th2 cells in asthmatic airways

Theophylline has been used in the treatment of asthma for many decades and is still used worldwide for the treatment of asthma. Low dose theophylline has recently been shown to have significant anti-inflammatory effects in the airways of the asthmatic patients [23,24]. This is supported by a reduced infiltration of eosinophils and CD4+ lymphocytes into the airways of asthmatic patients after allergen challenge subsequent to low doses of theophylline [25,26]. Low doses of theophylline also have reduced the number of CD4+ and CD8+ T lymphocytes and IL-4- and IL-5-containing cells in bronchial biopsies of asthmatic subjects [27,28]. In addition, in an uncontrolled study, in asthmatic patients, regular treatment with low doses of theohylline reduced sputum eosinophils and IL-5 expression, but not sputum CD4+ T lymphocytes and IFN-γ [29]. In patients with severe persistent asthma treated with high-doses of inhaled glucocorticoids, withdrawal of theophylline results in increased numbers of activated CD4+ cells and eosinophils in bronchial biopsies [30]. *In vitro*, low concentrations of theophylline (<25 nM) can inhibit the migration of T lymphocytes to chemotactic factors [31]. Furthermore, theophylline, at high concentrations, has been shown to reduce IL-2 production by T cells and IL-2-dependent T cell proliferation and induces nonspecific suppressor activity in human peripheral blood lymphocytes [32].

*In vitro*, high concentrations of theophylline suppress CD4+ expression of both Th1 and Th2, excluding IL-5, cytokines probably via inhibition of phosphodiesterases [33,34]. In an animal model of asthma, both low and high doses of aminophylline are effective in preventing late-phase bronchoconstriction, bronchial hyperresponsiveness, and airway inflammation. Furthermore, aminophylline decreases Th2-related cytokine mRNA expression but increases Th1-related cytokines mRNA expression [35].

## Effects of leukotriene-receptor antagonists and synthesis-inhibitors on Th2 cells in asthmatic airways

Leukotriene-receptor antagonists (pranlukast, zafirlukast, montelukast) and synthesis-inhibitors (zileuton) reduce the severity of bronchial hyperresponsiveness in asthma. In asthmatic patients, these agents can reduce sputum, bronchial biopsy and peripheral blood eosinophilia induced by experimental challenge with allergen, aspirin, sulfur dioxide or leukotriene (LT)E_4_[3,36].

*In vitro*, the cysteinyl-leukotriene receptor antagonist pranlukast can concentration-dependently inhibit the release of Th2 cytokines (IL-3, IL-4, GM-CSF), but not of the Th1 cytokine IL-2, from mite allergen-stimulated PBMCs from asthmatic patients [37]. Also, in an animal model of asthma, treatment with pranlukast reduces IL-5 but has no effect on IFN-γ production [38]. In contrast, high-doses of montelukast reduce IL-4, IL-5 and IL-13 levels in the lung and IL-4 and IL-5 expression in BAL [39]. In similar studies, montelukast decreases IL-4 mRNA expression in the lungs while increasing IFN-γ mRNA expression after allergen challenge [40]. Interestingly, the treatment of children with allergic rhinitis with montelukast induces a significant decrease of nasal lavage IL-4 and IL-13 and a significant increase of IFN-γ levels [41].

## Effects of β_2_-agonists on Th2 cells in asthmatic airways

Despite some positive *in vivo* studies, particularly with formoterol and more recently with salmeterol, the anti-inflammatory effect of short- and long-acting inhaled β_2_-agonists has not been convincingly demonstrated in asthmatic airways [3,8]. Although, it was initially proposed that the bronchodilating and symptom-relieving effects of long-acting inhaled β_2_-agonists may potentially mask increasing inflammation and delay awareness of worsening asthma, there is no evidence that long-acting inhaled β_2_-agonists worsen exacerbations of asthma or the chronic airway inflammation in asthma [3].

*In vitro* studies using resting and activated murine Th1 and Th2 cells have shown that a detectable level of the β2AR is expressed only on resting and activated Th1 cells, but not Th2 cells [42,43]. Baseline levels of intracellular cAMP are similar in both subsets, but β_2_-agonists induce an increase in cAMP levels in Th1 cells only [43].

Human peripheral blood mononuclear cells when stimulated in vitro with β_2_-agonists show decreased levels of IFN-γ and increased levels of IL-4, IL-5, and IL-10, an effect that is thought to be mediated by decreasing IL-12 production thereby suggesting that β_2_-agonists promote Th2 cytokine production. β_2_-agonists are potent and selective inhibitors of LPS- and CD40-CD40L-stimulated IL-12 production by human macrophages and dendritic cells [44]. In accord with their ability to suppress IL-12 production, when β_2_-agonists are added to neonatal cord blood T cells, they selectively inhibit the development of Th1 cells and enhance Th2 cell development [44]. However, in other *in vitro* studies β_2_-agonists have been shown to inhibit the secretion of IL-4 and IL-5 in T cell lines [45]. Regular treatment of patients with mild asthma with the long-acting β_2_-agonist formoterol does not decrease the number of IL-4 immunoreactive cells in their bronchial mucosa [46].

## Effects of cromoglycate and nedocromil on Th2 cells in asthmatic airways

Cromoglycate (cromolyn) has been shown to inhibit the IgE-mediated mediator release from human mast cells, and to suppress the activation of, and mediator release from, other inflammatory cells (macrophages, eosinophils, monocytes). Prolonged treatment of asthmatic patients with cromoglycate decreases the percentage of blood, sputum and BAL eosinophils, suggesting a direct anti-inflammatory effect in human asthmatic airways. Cromoglycate has also been shown to inhibit chloride channels *in vitro *[8].

Cromoglycate and nedocromil are both very well tolerated and still widely prescribed, in some countries, for the treatment of asthma in children. However the majority of controlled studies do not show any efficacy of these drugs in the treatment of persistent asthma compared with placebo although they show some efficacy in exercise-induced bronchoconstriction [8]. *In vitro* studies also suggest that nedocromil can modulate the differentiation of Th1/Th2 cells [47] however there is a complete absence of controlled clinical trials in asthmatic patients using these drugs measuring the Th1/Th2 balance in the lower airways.

## Omalizumab

There is a complete absence of controlled clinical trials in asthmatic patients using omalizumab measuring the Th1/Th2 balance in the lower airways.

## Effects of immunosuppressant drugs on Th2 cells in asthmatic airways

Methotrexate may have a small glucocorticoid sparing effect in adults with asthma who are dependent on oral glucocorticoids. However, the overall reduction in daily steroid use is probably not large enough to reduce steroid-induced adverse effects. This small potential to reduce the impact of steroid side-effects is probably insufficient to offset the adverse effects of methotrexate [2,48]. The absence of an inhibitory effect of methotrexate on a number of inflammatory cells in the blood and mucosa of the asthmatic patients suggests that the steroid-sparing effect of methotrexate is achieved by modulating cell function rather than cell number [8]. Cyclosporin A inhibits the allergen-induced late asthmatic reaction, the allergen-induced increase in IL-5 and GM-CSF in mRNA+ cells in BAL, and in the number of eosinophils in blood and bronchial mucosa, but not the early asthmatic reaction [8]. *In vitro* cyclosporin A inhibit allergen-driven T-cell proliferation, production of IL-2, IL-4, and IL-5 by human CD4+ helper T cells, and IL-5 production in PBMCs from allergen-sensitized atopic asthmatic individuals at physiologic concentrations. *In vitro* cyclosporin A, at putative therapeutic concentrations, has antiproliferative effects, with equivalent potency, on T-lymphocytes from glucocorticoid-sensitive and -resistant asthmatics but *in vitro* T-lymphocyte proliferation assays are not predictive of clinical response to cyclosporin therapy in chronic severe asthma [8,49].

In summary, the glucocorticoid sparing effect of cyclosporin A is small and of questionable clinical significance. Given the side effects of cyclosporin A, the available evidence does not recommend routine use of this drug in the treatment of oral glucocorticoid-dependent asthma [2,8,50].

## New drugs which can potentially interfere with Th2 cells in asthmatic airways

Many new drugs are now in development for the treatment of asthma. There has been an intensive search for anti-inflammatory treatments for bronchial asthma that are as effective as glucocorticoids but with fewer side effects. Whereas one approach is to seek glucocorticoids with a greater therapeutic index, other approaches involve developing different classes of anti-inflammatory drugs [51]. There is also a need for new treatments to deal with the small minority of patients with more severe asthma that is currently not well controlled by high doses of inhaled glucocorticoids and a need for a safe oral drug that would be effective in all atopic diseases (including asthma, allergic rhinitis and atopic dermatitis), as they often occur together [51].

Selective inhibition of Th2 lymphocytes function may be effective and well tolerated and there are active research programmes for such drugs in most pharmaceutical companies [52].

## Selective inhibitors of phosphodiesterase 4

A promising class of novel anti-inflammatory treatments for asthma are the selective inhibitors of phosphodiesterase 4 (PDE_4_). PDE4 is expressed in macrophages, neutrophils, T cells and airway smooth muscle cells [8]. These compounds inhibit the hydrolysis of intracellular cAMP, which may result in bronchodilation and suppression of inflammation. There are many compounds in this new class of drugs in clinical development; however most of the clinical studies reported have been performed with cilomilast and roflumilast [8]. There are controlled clinical trials suggesting some efficacy of roflumilast in mild to moderate asthma and to prevent exercise-induced asthma in adults [53]. However, the development of cilomilast as an antiasthma drug has apparently been suspended.

There are no significant differences in the expression of PDE4A, PDE4B and PDE4D in peripheral blood CD4 and CD8 lymphocytes from normal and asthmatic patients [54]. PDE4 subtype expression is lower and shows more intersubject variability in CD8+ cells however [54]. Furthermore, *in vitro*, Th1 lymphocytes show a reduced expression of PDE4C isoform and a lack of PDE4D isoform compared to Th2 lymphocytes [55].

Cyclic adenosine monophosphate (cAMP) is a negative regulator of T-cell activation. However, the effects of cAMP on signaling pathways that regulate cytokine production and cell cycle progression in Th1 and Th2 lymphocytes remain controversial.

*In vitro,* using allergen-induced human Th1 and Th2 clones both Th1 and Th2 cytokines production are equally inhibited by selective PDE4 inhibitors [55]. However, the increase in intracellular cAMP is significantly more in Th2 compared with Th1 clones [55]. *In vitro*, selective PDE4 inhibitors inhibit proliferation and IL-4 and IL-5 secretion by human allergen-specific Th2 lymphocytes and Th1 and Th2 clones [34,56]. Other *in vitro* studies suggest that PDE4 inhibitors have complex inhibitory effects on Th1-mediated immunity at the concentration ranges achievable *in vivo*, whereas Th2-mediated responses are mostly unaffected or even enhanced [57]. The Th2 skewing of the developing immune response is explained by the effects of PDE inhibitors on several factors contributing to T cell priming: the cytokine milieu; the type of costimulatory signal, i.e., up-regulation of CD86 and down-regulation of CD80; and the antigen avidity [57].

In animal studies, PDE4 inhibitors inhibit antigen-mediated T cell proliferation and skew the T cell cytokine profile toward a Th2 phenotype by downregulating the expression or production of Th1 cytokines without affecting Th2 cytokine expression [58,59].

There is a complete absence of controlled clinical trials in asthmatic patients using these drugs measuring the Th1/Th2 balance in the lower airways.

## Chemokine receptors antagonists targeting Th2 cells in asthmatic airways

Numerous antibodies, receptor blocking mutant chemokines and small molecules are now being evaluated for the treatment of asthma. Chemokines have proven to be amenable drug targets for the development of low molecular weight antagonists by the pharmaceutical industry. CCR3, CCR4, CCR8, and CRTH2 non-peptide antagonists are involved in the recruitment and/or activation of Th2 cells in the lung and are now being evaluated for the treatment of bronchial asthma but so far, no clinical data for these compounds have been reported. However, over the next few years it is expected that many studies will have been published at which time the potential of these exciting new targets will be fully realized [60,61].

## CCR3 antagonists and asthma

A range of low molecular weight chemicals have been developed to antagonise CCR3, with the aim of selectively inhibiting eosinophil recruitment into tissue sites. However, the results of recent clinical trials with monoclonal antibodies directed against IL-5 question the role of eosinophils in mediating the symptoms of asthma [62]. For this reason, the plans for clinical development of many CCR3 antagonists in asthma have been put on hold [63].

More recently novel oral CCR3 selective antagonists have been developed by many pharmaceutical companies including Brystol-Myers Squibb, GSK and Yamanouchi Pharmaceuticals [64-71], including double CCR3 and H1 receptor antagonists [72]. Some of these compounds are now undergoing clinical trials in asthma.

These compounds are able to prevent the activation and recruitment of eosinophils, but not lymphocytes, in animal models of asthma [66,71,73]. However, in another animal model of asthma, a CCR3 antagonist did not decrease the number of eosinophils in lung tissues but only antigen-induced clustering of eosinophils along the airway nerves [74]. Immunostaining shows eotaxin in airway nerves and in cultured airway parasympathetic neurons [74]. *In vitro* both IL-4 and IL-13 increase expression of eotaxin in airway parasympathetic neurons [74]. Thus, signaling via CCR3 mediates eosinophil recruitment to airway nerves and may be a prerequisite to blockade of inhibitory muscarinic M2 receptors by eosinophil major basic protein [74].

N-nonanoyl (NNY)-CCL14[10-74] (NNY-CCL14) is an N-terminally truncated and modified peptide derived from the chemokine CCL14 that in vitro inhibits the activity of CCR3 on human eosinophils, because it is able to induce internalization of CCR3 and to desensitize CCR3-mediated intracellular calcium release and chemotaxis. In contrast to naturally occurring CCL11, NNY-CCL14 is resistant to degradation by CD26/dipeptidyl peptidase IV (DP4). This compound is effective in animal models of asthma [75]. N-Nonanoyl-CCL11 (NNY-CCL11) represents another similar compound with dual activity restricted to CCR3 and CCR5. It also has receptor-inactivating capacity and stability against DP4 degradation [76]. All these new compounds have been developed by Ipf Pharmaceuticals ().

Specific targeting of inhibitory receptors on CCR3+ cells may be an alternative approach. For example cross-linking of inhibitory receptor protein 60 (IR-p60)/CD300a inhibits mast cell and eosinophil activation and co-aggregation of CD300a with CCR3 using a bi-specific antibody fragment (LC1) has been shown to be effective in an animal model of asthma [77], but is still untested in human asthma.

TPIASM8 is a new inhaled compound consisting of two modified RNA-targeting oligonucleotides directed against the CCR3 receptor, and the common β subunit for the receptors of IL-3, 5 and GM-CSF. TPIASM8 is currently in phase II clinical development for the treatment of asthma (). This novel approach is expected to have advantages over single mediator antagonists.

## CCR4 antagonists and asthma

The utility of developing CCR4 antagonists is controversial because CCR4-deficient mice do not show any change in cell recruitment in the lung or induction of airway hyperresponsiveness [78]. However many CCR4 antagonists are now in preclinical development and have been shown to be effective in reducing the chemotaxis of Th2 cells *in vitro* and lung eosinophilic inflammation in an animal model of asthma [79-84]. There are no published controlled clinical trials of these compounds in asthma.

## CCR8 antagonists and asthma

The *in vivo* role of the CCL1/CCR8 axis in Th2-mediated inflammation is far from clear. CCR8-deficient mice have a marked reduction of airway eosinophil infiltration and allergen-induced airway hyperresponsiveness, but the CCR8 is not essential for the development of airway inflammation in other animal models of asthma [85,86]. Overall these data, whilst highlighting a potential major role for CCR8, suggest that multiple chemokines and chemokine receptors may have redundant functions in the pathogenesis of bronchial asthma. CCR8 and CCL1, the CCR8 ligand, antagonists have been recently developed [87-90]. There are no published controlled clinical trials of these compounds in asthma.

## CRTH2 antagonists and asthma

Ramatroban (Baynas, BAY u3405), an orally active, tromboxan (Tx)A2 antagonist marketed in Japan for the treatment of allergic rhinitis, is also an antagonist for CRTH2, and i*n vitro* inhibits PGD2-induced migration of eosinophils. Ramatroban has been shown to partially attenuate prostaglandin PGD2-induced bronchial hyperresponsiveness in humans, as well as reduce antigen-induced early- and late-phase inflammatory responses in animal models of asthma [91].

A new selective CRTH2 antagonist named TM30089 is structurally closely related to ramatroban but with less affinity for TP and many other receptors including the related anaphylatoxin C3a and C5a receptors, selected chemokine receptors and the cyclooxygenase isoforms 1 and 2, attenuates airway eosinophilia and mucus cell-hyperplasia in an animal model of asthma [92].

Many novel selective orally active CRTH2 antagonists have been recently developed [93-99], but there are no published studies on the effect of CRTH2 antagonists in asthmatic patients, the results of the ongoing clinical trials are awaited with interest [97]. A once day oral molecule ODC9101 is now in phase IIa clinical trials in asthma ().

## CCR5 agonists and asthma

Aminooxypentane (AOP)-RANTES/CCL5 is a full agonist of human CCR5 [100] a chemokine receptor expressed selectively on human Th1 lymphocytes. In an animal model of asthma AOP-RANTES/CCL5 decreases allergen-induced airway inflammation suggesting that targeting CCR5 may also be effective [100].

## Sphingosine 1-phosphate receptor agonists

Sphingosine 1-phosphate (S1P) in blood, lymph, and immune tissues stimulates and regulates T cell migration through their S1P(1) (endothelial differentiation gene encoded receptor-1) G protein-coupled receptors (S1P1Rs). S1P1Rs also mediate suppression of T cell proliferation and cytokine production. In fact S1P decreases CD4 T cell generation of IFN-γ and IL-4 [101].

The novel oral immunomodulator FTY720 is a chemical derivative of myriocin, a metabolite of the ascomycete Isaria sinclairii. The drug has recently been shown to be effective in human kidney transplantation. In contrast to classical immunosuppressants such as cyclosporine A or FK506, FTY720 selectively and reversibly sequesters lymphocytes but not monocytes or granulocytes from blood and spleen into secondary lymphoid organs, thereby preventing their migration toward sites of inflammation and allograft rejection. Moreover, FTY720 does not impair T cell activation, expansion, and memory to systemic viral infections or induce T cell apoptosis at clinically relevant concentrations [102]. FTY720 is a structural analog of sphingosine and following *in vivo* phosphorylation acts as a agonist at S1P1Rs to block cell motility [102]. This leads to sequestration of lymphocytes in secondary lymphatic tissues and thus away from inflammatory lesions. Both Th1 and Th2 cells express a similar pattern of FTY720-targeted S1P1Rs. The inhibitory effect of FTY720 on airway inflammation, airway hyperresponsiveness, and goblet cell hyperplasia in an animal model of asthma, suggests a potential role of this compound in the treatment of asthma [102]. The accompanying lymphopenia could be a serious side effect that would preclude the use of oral FTY720 as an antiasthmatic drug [103].

However, in an animal model of asthma inhalation of FTY720 prior to or during ongoing allergen challenge suppresses Th2-dependent eosinophilic airway inflammation and bronchial hyperresponsiveness without causing lymphopenia and T cell retention in the lymph nodes [103].

Local treatment with FTY720 inhibits migration of lung dendritic cells to the mediastinal lymph nodes, which in turn inhibited the formation of allergen-specific Th2 cells in lymph nodes. Also, FTY720-treated dendritic cells are less potent in activating naive and effector Th2 cells [103].

## Ca2+ release-activated Ca2+ channels blockers and asthma

The pyrazole derivative, YM-58483 (BTP2; ), potently inhibits Ca2+ release-activated Ca2+ (CRAC) channels and IL-2 production in T cells and IL-4 and IL-5 production in stimulated murine Th2 cells, and IL-5 production in stimulated human whole blood cells. YM-58483 inhibited airway eosinophil infiltration, IL-4 and cysteinyl-leukotrienes content and late phase asthmatic bronchoconstriction in animal models of asthma [104]. There are no published studies on human asthma using CRAC channel inhibitors.

## Transcription factor modulators

Asthma is characterized by an increased expression of components of the inflammatory cascade. These inflammatory proteins include cytokines, chemokines, growth factors, enzymes, receptors and adhesion molecules. The increased expression of these proteins seen in asthma is the result of enhanced gene transcription since many of the genes are not expressed in normal cells but are induced in a cell-specific manner during the inflammatory process. Changes in gene transcription are regulated by transcription factors, which are proteins that bind to DNA and modulate the transcriptional apparatus. Transcription factors may therefore play a key role in the pathogenesis of asthma [105,106].

Many transcription factors (for example NF-κB, AP-1, GATA-3, STAT-1 STAT-6, c-Maf, NFATs and SOCS) have been implicated in the differentiation of Th2 lymphocytes and therefore represent therapeutic targets for asthma.

Several new compounds based on interacting with specific transcription factors or their activation pathways are now in development for the treatment of asthma and some drugs already in clinical use (such as glucocorticoids) work via transcription factors ([7]. One concern about this approach is the specificity of such drugs, but it is clear that transcription factors have selective effects on the expression of certain genes and this may make it possible to be more selective [105,106]. In addition, there are cell specific transcription factors that may be targeted for inhibition, which could provide selectivity of drug action. One such example is GATA-3, which has been reported to have a restricted cellular distribution. In asthma it may be possible to target drugs to the airways by inhalation, as is currently utilised for inhaled glucocorticoids to avoid, or minimize, any systemic effects [105,106].

## NF-κB and AP-1

Transcription factors, such as nuclear factor (NF)-κB and activator protein (AP)-1, play an important role in the orchestration of the airway inflammation in asthma. The role of NF-κB and AP-1 should be seen as an amplifying and perpetuating mechanism that will exaggerate the disease-specific inflammatory process. *In vitro*, AP-1 and NF-κB are also important for the function of Th2 cells [105,106]. There is evidence for activation of both NF-κB and AP-1 in the bronchial epithelial cells of patients with asthma [105,106]. There are several possible approaches to the inhibition of NF-κB. The most promising approach might be the inhibition of IKKβ by small-molecule inhibitors, which are now in development by several companies [105-108]. Alternative strategies involve the development of small peptide inhibitors of IKKβ/IKKγ association. Interestingly, in animals NF-κB decoy oligodeoxynucleotides prevent and treat oxazolone-colitis and thus affect a Th2-mediated inflammatory process [109]. One concern about the long-term inhibition of NF-κB is that effective inhibitors may cause side effects, such as increased susceptibility to infections, as mice that lack NF-κB genes succumb to septicaemia [105-108].

A small-molecule inhibitor, PNRI-299, that targeting the oxidoreductase redox effector factor-1, selectively inhibits AP-1 transcription, without affecting NF-κB transcription, significantly reduces airway eosinophil infiltration, mucus hypersecretion, edema, and lung IL-4 levels in a mouse asthma model [110]. In an animal model of asthma intratracheally delivered AP-1 decoy oligodeoxynucleotides attenuate eosinophilic airway inflammation, airway hyperresponsiveness, mucous cell hyperplasia, production of allergen-specific immunoglobulins, and synthesis of IL-4, IL-5, and IL-13 in the lung [111].

## GATA-3

The transcription factor GATA-3 seems to be of particular importance in the differentiation of human Th2 cells and its expression is increased in the peripheral venous blood T cells from atopic asthmatics [106] and in bronchial biopsies of stable asthmatics compared to controls and in BAL cells of asthmatics after allergen challenge [112,113].

Many studies indicate a critical role for GATA-3 in the development of airway eosinophilia, mucus hypersecretion and airway hyperesponsiveness in animal models of asthma [114] and suggest that local delivery of GATA-3 antisense oligonucleotides may be a novel approach for the treatment of asthma [115]. This approach has the potential advantage of suppressing the expression of various proinflammatory Th2 cytokines simultaneously rather than suppressing the activity of a single cytokine.

## STAT1 blockers and asthma

The intracellular signaling intermediate signal transducer and activator of transcription (STAT)1 mediates many effects of IFN-γ and is implicated in the activation of T-bet, a master regulator of Th1 differentiation. In animal models Th1 and Th2 cell trafficking is differentially controlled *in vivo* by STAT1 and STAT6, respectively. STAT6, which regulates Th2 cell trafficking, had no effect on the trafficking of Th1 cells and STAT1 deficiency does not alter Th2 cell trafficking [116]. STAT1 in peripheral tissue regulates the homing of antigen-specific Th1 cells through the induction of a distinct subset of chemokines (CXCL9, CXCL10, CXCL11, and CXCL16) [116]. CXCL10 replacement partially restored Th1 cell trafficking in STAT1-deficient mice i*n vivo*, and deficiency in CXCR3, the receptor for CXCL9, CXCL10, and CXCL11, impaired the trafficking of Th1 cells [116]. 

STAT1 expression and activation is elevated in asthmatic bronchial epithelial cells in some, but not all [117], studies [118]. This has led to the development of decoy oligonucleotides designed to block STAT1 activity. In an animal model of asthma a single application of this STAT1 decoy oligonucleotides significantly reduces airway hyperresponsiveness, the number of BAL eosinophils and lymphocytes and the BAL level of IL-5 [119]. This decoy oligonucleotides designated AVT-01 is currently undergoing phase II studies in asthmatic patients ().

## STAT-6

STAT6-knockout animals do not express Th2-type chemokines in the lung and as a result do not recruit allergen-specific Th2 cells into the lung following allergen challenge [120]. Furthermore, STAT6-knockout animals fail to develop goblet cell metaplasia in response to IL-13 instillation, and this response can be rescued by epithelial-directed expression of a STAT-6 transgene [121]. Previous data investigating the localisation of STAT6 in the airways of man has produced divergent results. In two studies STAT-6 is present only within infiltrating cells of the nose and bronchial mucosa [122,123], whilst in another two studies STAT-6 is expressed predominantly within the bronchial epithelium of mild asthmatic subjects [124,125]. Therefore, although tempting as a target, a clear rationale for targeting STAT-6 in asthma is not currently available.* In vitro* a STAT6 selective antisense significantly reduces eotaxin release from human airway smooth muscle stimulated by IL-13 or IL-4 [126]. Interestingly, in an animal model of asthma niflumic acid, a relatively specific blocker of calcium-activated chloride channel, inhibits IL-13-induced goblet cell hyperplasia, MUC5AC expression, airway hyperresponsiveness, BAL eosinophilia and eotaxin increase. Niflumic acid also inhibits STAT6 activation and eotaxin expression in bronchial epithelial cells *in vitro *[127].

The adipocyte/macrophage fatty acid–binding protein (FABP) aP2 is expressed in bronchial epithelial cells and it is strongly upregulated by both IL-4 and IL-13 in a STAT6-dependent manner. The presence of functional aP2 has been shown very important in an animal model of asthma [128].

## c-maf

The effects of c-MAF appear to be fairly selective, since *in vitro* studies have demonstrated that this factor is critical for the production of IL-4, but not for IL-5 or IL-13 [129,130]. c-Maf expression in T lymphocytes is regulated by IL-4 levels during Th differentiation. ICOS costimulation potentiates the TCR-mediated initial IL-4 production, possibly through the enhancement of NFATc1 expression [131]. In animals c-maf is a Th2 cell-specific transcription factor, which promotes the differentiation of Th2 cells mainly by an IL-4-dependent mechanism [132]. c-maf-transgenic mice produce higher serum levels of IgE and IgG1, and their Th cells spontaneously developed into Th2 cells *in vitro*[133]. In contrast, c-maf-deficient (c-maf ^-^/_-_) Th cells are unable to differentiate into Th2 cells in the absence of exogenous IL-4. Although c-maf ^-^/_-_Th2 cells, differentiated in the presence of exogenous IL-4, produced normal levels of IL-5, IL-10, and IL-13, the production of IL-4 is severely impaired [129]. Furthermore, c-maf, independent of IL-4, is also essential for normal induction of CD25 (IL-2Rα chain) in developing Th2 cells, which express higher levels than seen in Th1 cells. Blockade of IL-2R signaling selectively inhibits the production of Th2 cytokines, but not IFN-γ or IL-2 [132]. An increased number of c-maf immunoreactive cells have been observed within the sputum and bronchial biopsies of asthmatic patients compared with control subjects [122,134]. There are no published studies on the effect of selective c-maf inhibitors *in vitro and/or in vivo*.

## NFATs

Nuclear factor of activated T cells (NFAT) was originally described as a T-cell-specific transcription factor, which is expressed in activated, but not resting T cells and is required for IL-2 gene transcription. However, we now know that NFAT is not T cell specific but is also expressed in many other types of cells (e.g. mast cells, monocytes, macrophages, eosinophils, epithelial cells, smooth muscle and endothelial cells) [135,136].

The NFAT family of transcription factors include the cytoplasmic NFAT transcription factors [NFATc1 (NFATc), NFATc2 (NFATp), NFATc3 (NFAT4, NFATx), NFATc4 (NFAT3), NFATc5] and nuclear NFAT (NFATn). NFATc proteins are localised in the cytoplasm and activated by stimulation of receptors coupled to calcium mobilisation. Receptor stimulation and calcium mobilisation result in activation of many intracellular enzymes, including the calcium and calmodulin dependent phosphatase calcineurin, a major upstream regulator of NFATc proteins. Stimuli that elicit calcium mobilisation result in the rapid dephosphorylation of NFATc proteins and their translocation to the nucleus where they have strong binding affinity to DNA [137,138].

NFATs are ubiquitous regulators of cell differentiation and adaptation [135] but in stimulated T cells NFATs are mainly involved in the regulation of proliferation and Th1/Th2 cytokine production [139,140]. For instance the GM-CSF enhancer contains four composite NFATs/AP-1 DNA binding sites, three of which demonstrate cooperative binding of NFATs and AP-1. The fourth site binds NFATs and AP-1 independently. NFATs show a characteristic ability to interact with AP-1 and NF-κB DNA binding and transactivation. It has been shown that coupled NFAT:AP-1 is more stable and has higher affinity for DNA. Interestingly, preferential activation of NFATc1 correlates with mouse strain susceptibility to allergic responses and IL-4 gene expression [141]. NFATc2 appears to be important for the activation of the Th2 cells [142-147]. In contrast, NFATc3 seems to enhances the expression of the Th1 cytokine genes, IFN-γ and TNF-α, and to suppress Th2 cytokine genes such as IL-4 and IL-5 in Th2 cells [148,149].

As substrates for calcineurin, NFATs proteins are major targets of the immunosuppressive drugs cyclosporin A (see above) and FK506 because of their ability to inhibit dephosphorylation of NFATs. Bis(trifluoromethyl)pyrazoles (BTPs) are novel inhibitors of both Th1 and Th2 cytokines production [150,151]. Identified initially as inhibitors of IL-2 synthesis, BTPs inhibit IL-2 production with a 10-fold enhancement over cyclosporin A. Additionally, the BTPs show inhibition of IL-4, IL-5, IL-8, and CCL11 production [150,151]. Unlike the IL-2 inhibitors, cyclosporin A and FK506, the BTPs do not directly inhibit the dephosphorylation of NFAT by calcineurin. There are no published studies on NFATc1 inhibitors in asthma.

## SOCS modulation of Th1/Th2 differentiation

Suppressors of cytokine signaling (SOCS)-1 interacts directly with the Janus kinases (JAK) and inhibits their tyrosine-kinase activity [152]. SOCS1 is an important *in vivo* inhibitor of type I interferon signaling [153]. A SOCS1 promoter polymorphism (-1478CA>del) is associated with adult asthma [153]. *In vitro* this SNPs enhances the transcription of SOCS1 in human lung epithelial cells, but reduces phosphorylation of STAT1 stimulated with IFN-β [153]. SOCS-3 is predominantly expressed in Th2 cells and inhibits Th1 differentiation [154]. SOCS3 also has a role in Th3 differentiation [155,156]. SOCS-3 transgenic mice shows increased Th2 responses and an asthma-like phenotype. In contrast, SOCS-3 knockout mice, has decreased Th2 development [157]. These data suggest that SOCS-3 may be a new target for the development of antiasthma drugs [156]. It has been suggested that enhancement of the expression of SOCS-5 in CD4^+^ T cells might be a useful therapeutic approach to Th2-dominant diseases [158]. In fact, transfer of primed CD4^+^ T cells constitutively expressing SOCS-5 along with eye drop challenges in a murine allergic conjunctivitis model resulted in attenuated eosinophilic inflammation with enhanced IFN-γ and decreased IL-13 production [159]. However, it should be noted that SOCS-5 appears to be dispensable for the development of Th1 responses *in vivo*, as demonstrated by use of the SOCS-5 knockout mice [160]. SOCS-5-deficient CD4^+^ T cells can differentiate into either Th1 or Th2 cells with the same efficiency [160]. These data have been confirmed, in an animal model of asthma where significantly more eosinophils in the airways and higher BAL levels of IL-5 and IL-13 were observed in the SOCS-5 transgenic than the wild-type mice. Airway hyperresponsiveness in the asthma model of SOCS-5 transgenic was also enhanced compared to wild-type mice. Ovalbumin-stimulated CD4^+^ T cells from the primed SOCS-5 transgenic mice produced significantly more IL-5 and IL-13 than CD4^+^ T cells from wild-type mice [161]. This finding raises questions about the therapeutic utility of using enhancement of SOCS-5 expression for Th2-mediated diseases, such as asthma.

## Peroxisome proliferator-activated receptors

Peroxisome proliferator-activated receptors (PPARs) are transcription factors belonging to the nuclear receptor superfamily. PPARs are activated by an array of polyunsaturated fatty acid derivatives, oxidized fatty acids, and phospholipids and are proposed to be important modulators of allergic inflammatory responses [162]. The three known PPAR subtypes α, γ and δ, show different tissue distributions and are associated with selective ligands. PPARs are expressed by eosinophils, T-lymphocytes and alveolar macrophages, as well as by epithelial, and smooth muscle cells. PPAR-α and -γ are expressed in eosinophils and their activation inhibits *in vitro* chemotaxis and antibody-dependent cellular cytotoxicity [163]. PPAR-α and -γ are both expressed in monocytes/macrophages. PPAR-γ is expressed in eosinophils and T lymphocytes. *In vivo*, inflammation induced by leukotriene B_4_ (LTB_4_), a PPAR-α ligand, is prolonged in PPAR-α-deficient mice, suggesting an anti-inflammatory role for this receptor [164]. In contrast, in mice injected with lipopolysaccharide (LPS), activation of PPAR-α induces a significant increase in plasma tumour necrosis factor- (TNF-α) levels [164].

PPAR-γ ligands significantly inhibit production of IL-5 from T cells activated *in vitro*[165]. In a murine model of allergic asthma, mice treated orally with ciglitazone, a potent synthetic PPAR-γ ligand, have significantly reduced lung inflammation and mucous production following induction of allergic asthma. T cells from these ciglitazone treated mice also produce less IFN-γ, IL-4, and IL-2 upon rechallenge in vitro with allergen [165].

Activation of PPAR-γ alters the maturation process of dendritic cells (DCs), the most potent antigen-presenting cells. By targeting DCs, PPAR-γ activation may be involved in the regulation of the pulmonary immune response to allergens [162]. Using a model of sensitization, based on the intratracheal transfer of ovalbumin-pulsed DCs, rosiglitazone, another selective PPAR-γ agonist, reduces the proliferation of antigen-specific T cells in the draining mediastinal lymph nodes but dramatically increases the production of IL-10 by these T cells. After aerosol challenge, the recruitment of BAL eosinophils is strongly reduced compared to control mice. Inhibition of IL-10 activity with anti-IL-10R antibodies partly restored the inflammation [162,166].

PPAR-α and PPAR-γ ligands also decrease antigen-induced airway hyperresponsiveness, lung inflammation and eosinophilia, cytokine production, and GATA-3 expression as well as serum levels of antigen-specific IgE in many different animal models of asthma [163,167-170]. These studies suggest that PPAR-α and PPAR-γ (co)agonists might be a potential anti-inflammatory treatment for asthma [171-173]. Interestingly, *in vitro* theophylline, procaterol and dexamethasone induce PPAR-γ expression in human eosinophils [174,175].

## MAP kinase inhibitors

There are three major mitogen-activated protein (MAP) kinase pathways and there is increasing recognition that these pathways are involved in the pathogenesis of asthma.

## p38 MAPK inhibitors

p38 MAPK kinase is a Ser/Thr kinase involved in many processes thought to be important in lower airways inflammatory responses and tissue remodeling. There is, however, a paucity of reports specifically addressing the role of p38 kinase in asthma [107,176].

There are four members of the p38 MAP kinase family and they differ in their tissue distribution, regulation of kinase activation and subsequent phosphorylation of downstream substrates. They also differ in terms of their sensitivities toward the p38 MAP kinase inhibitors [107,176]. In general, p38 MAPKs are activated by many stimuli, including cytokines, hormones, ligands for G protein-coupled receptors, and elevated levels of these cytokines are associated with asthma. The synthesis of many inflammatory mediators such as TNFα, IL-4, IL-5, IL-8, RANTES and eotaxins, thought to be important in asthma pathogenesis, are regulated through activation of p38 MAPK. p38 MAPK can affect the transcription of these genes but also has major effects on mRNA stability. In addition, p38 MAPK appears to be involved in glucocorticoid-resistance in asthma [107,176].

SB 203580, an early selective inhibitor of p38 MAP kinase, inhibits the synthesis of many inflammatory cytokines, chemokines and inflammatory enzymes. Interestingly, *in vitro* SB203580 appears to have a preferential inhibitory effect on synthesis of Th2 compared to Th1 cytokines, indicating their potential application in the treatment of asthma [177]. Inhaled p38α MAPK antisense oligonucleotide attenuates asthma in an animal model [178]. Several oral and inhaled p38MAPK inhibitors are now in clinical development [179]. Whether this new potential class of anti-inflammatory drugs will be safe in long-term studies in human asthma remains to be established. For the successful use of MAPK inhibitors in clinical trial on patients with asthma these compounds must be very specific to reduce the side-effects of the plethora of physiological MAPK functions. However, options to improve safety include inhaled delivery and use as a steroid-sparing agent.

## JNKs

The c-Jun NH_2_-terminal kinases (JNKs) phosphorylate and activate members of the activator protein-1 (AP-1) transcription factor family and other cellular factors implicated in regulating altered gene expression, cellular survival (apoptosis), differentiation and proliferation in response to cytokines, growth factors and oxidative stress and cancerogenesis. Since many of these are common events associated with the pathogenesis of asthma, the potential of JNK inhibitors as therapeutics has attracted considerable interest. Furthermore, in patients with severe glucocorticoid-resistant asthma there is increased expression of the components of the pro-inflammatory transcription factor activator protein 1 (AP-1) and enhanced JNK activity [11,180].

The c-jun N-terminal (JNK) group of MAPK consists of three isoforms, encoded by three different genes, of which the JNK1 and 2 isoforms are widely distributed, while JNK3 is mainly located in neuronal tissue. Gene disruption studies in mice demonstrate that JNK is essential for TNFα-stimulated c-Jun phosphorylation and AP-1 activity, and is also required for some forms of stress-induced apoptosis. JNKs enhance the transcriptional activity of AP-1 by phosphorylation of the AP-1 component c-Jun on serine residues 63 and 73 and thereby increasing AP-1 association with the basal transcriptional complex. JNKs may also enhance the activity of other transcription factors such as ATF-2, Elk-1 and Sap-1a. Many immune and inflammatory genes including cytokines, growth factors, cell surface receptors, cell adhesion molecules and proteases such as matrix metalloprotease 1 (MMP-1) are regulated by AP-1 and ATF-2 presumably through the JNK pathway. JNKs do not only affect transcription of cytokine mRNAs but may also enhance the stability of some mRNAs such as that for IL-2 and nitric oxide synthase 2 (NOS2) [107].

JNK activation may also be important in the regulation of the immune response. JNK polarizes the differentiation of CD4+ T cells to a Th1-type immune response by a transcriptional mechanism involving the transcription factor nuclear factor of activated T cells 1 (NFATc1). JNK1 and JNK2 knockout mice have similar phenotypes but some subtle differences exist e.g. JNK2(-/-) CD8+ cells show enhanced proliferation whereas JNK1(-/-) CD8+ cells cannot expand [107].

SP600125 (Signal Pharmaceuticals/Celgene), a JNK inhibitor, inhibited TNFα and IL-2 production in human monocytes and Jurkat cells respectively and attenuated TNFα- and IL-1β-induced GM-CSF, RANTES and IL-8 production in primary human airway smooth muscle cells. In addition, in an animal model of chronic asthma SP-600125 (30mg/kg sc) reduces bronchoalveolar lavage accumulation of eosinophils and lymphocytes, cytokine release, serum IgE production and smooth muscle proliferation after repeated allergen exposure. Similar results were seen with the dual AP-1/NF-κB inhibitor SP100030 [181]. These data indicate that JNK inhibitors may be effective in the treatment of asthma.

A more selective second generation JNK-selective inhibitor [JNK-401(CC-401)] has successfully completed a phase I, double-blind, placebo controlled, ascending single intravenous dose study in healthy human volunteers (). Studies will examine whether JNK-401 will be glucocorticoid sparing and lacking many of the glucocorticoid side effects in humans.

The JNK pathway is implicated in a number of physiological and pathological functions in a range of human diseases. Due to the extensive cross-talk within this signalling cascade, as well as its cell-type- and response-specific modulation, it is difficult to predict potential adverse events that might arise from pathway inhibition. However, the fact that JNK inhibitors are progressing in clinical trials indicates that the utility of targeting this pathway for therapeutic benefit in asthma and will probably be determined within the near future.

## Heparin-like molecules

Glycosaminoglycans are large, polyanionic molecules expressed throughout the body. The GAG heparin, co-released with histamine, is synthesised by and stored exclusively in mast cells, whereas the closely related molecule heparan sulphate is expressed, as part of a proteoglycan, on cell surfaces and throughout tissue matrices [182]. An important feature of chemokines is their ability to bind to the glycosaminoglycan side chains of proteoglycans, predominately heparin and heparan sulfate. To date, all chemokines tested bind to immobilised heparin *in vitro*, as well as cell surface heparan sulfate *in vitro* and *in vivo*. These interactions play an important role in modulating the action of chemokines by facilitating the formation of stable chemokine gradients within the vascular endothelium and directing leukocyte migration, by protecting chemokines from proteolysis, by inducing chemokine oligomerisation, and by facilitating transcytosis [183]. There are data suggesting a role for mast cell-derived heparin in enhancing eotaxin-mediated eosinophil recruitment thereby reinforcing Th2 polarisation of inflammatory responses [183]. However, heparan sulfate has been shown *in vitro* to promote a Th1 response, decreasing the production of IL-4 [184]. Heparin and related molecules have been found to exert antiinflammatory effects *in vitro* and in animal models of asthma and that the antiinflammatory activities of heparin are dissociable from its anticoagulant nature, suggesting that these characteristics could yield novel antiinflammatory drugs for asthma [185-187]. The inhalation of heparin prevents exercise-induced bronchoconstriction [188-190]. A phase II study in mild asthma using IVX 0142, a novel heparin-derived oligosaccaride, has just been completed (.

## Modulators of the synthesis or action of key proinflammatory Th2 cell cytokines

Over one hundred mediators have now been implicated in asthmatic inflammation, including multiple cytokines, chemokines and growth factors. Blocking a single mediator is therefore unlikely to be very effective in this complex disease and mediator antagonists have so far not proved to be very effective compared with drugs that have a broad spectrum of anti-inflammatory effects, such as glucocorticoids [191]. The potential of blocking Th2 cytokines with pro-inflammatory action such as of IL-4, IL-5 and IL-13 has still not been completely explored. Also, anti-inflammatory cytokines such as IL-10, IL-12, IL-18, IL-21, IL-23, IL-27 and interferons may have a therapeutic potential in asthma. TNF-α blockers may also be useful particularly in severe asthma.

## IL-4 blockers and asthma

IL-4 analogues that act as antagonists have been developed which fail to induce signal transduction and block IL-4 effects *in vitro*. These IL-4 antagonists prevent the development of asthma *in vivo* in animal models [192,193].

However, the development of pascolizumab (SB 240683), a humanized anti-interleukin-4 antibody, as well as of a blocking variant of human IL-4 (BAY36-1677) has apparently been discontinued.

Soluble IL-4 receptors (sIL-4R) that act as IL-4R antagonists have also been developed [194], they are effective in an animal model of asthma [195] and a single nebulised dose of sIL-4R prevents the fall in lung function induced by glucocorticoid withdrawal in moderate/severe asthmatics [196]. Subsequent studies have shown that weekly nebulisation of sIL-4R improves asthma control over 3 months [197]. However further studies in patients with milder asthma proved disappointing and the clinical development of this compound has now been discontinued.

In an animal model of asthma a IL-4Rα antisense oligonucleotide (IL-4Rα ASO) specifically inhibits IL-4Rα protein expression in lung after inhalation in allergen-challenged mice [198]. Inhalation of IL-4Rα ASO attenuated allergen-induced AHR, suppressed airway eosinophilia and neutrophilia, and inhibited production of airway Th2 cytokines and chemokines in previously allergen-primed and -challenged mice [198]. Histological analysis of lungs from these animals demonstrated reduced goblet cell metaplasia and mucus staining that correlated with inhibition of MUC5AC gene expression in lung tissue. These data support the potential utility of a dual IL-4 and IL-13 oligonucleotide inhibitor in asthma, and suggest that local inhibition of IL-4Rα in the lung is sufficient to suppress allergen-induced pulmonary inflammation and AHR in mice [198].

A novel approach is represented by an IL-4 peptide-based vaccine for blocking IL-4 on a persistent basis. Vaccinated mice produces high titers of IgG to IL-4. Serum ovalbumin-specific IgE, eosinophil accumulation in BAL, goblet cell hyperplasia, tissue inflammation and AHR are markedly suppressed in vaccinated mice in an animal model of asthma [199].

## IL-13 blockers and asthma

Blocking IL-13, but not IL-4, in animal models of asthma prevents the development of airway hyperresponsiveness after allergen, despite a strong eosinophilic response [121,200,201]. In addition, soluble IL-13Rα2 is effective in blocking the actions of IL-13, including IgE production, pulmonary eosinophilia and airway hyperresponsiveness in animal models of asthma [202] and humanised IL-13Rα2 is now entering phase I clinical trials in asthma [203]. Also an anti-IL-13Rα1 antibody is in preclinical development for the treatment of asthma ().

A human anti human IL-13 IgG4 monoclonal antibody (CAT-354) that blocks IL-13 effects in an animal model of asthma [204] is in phase II clinical trials in severe asthma (). In addition, Centocor () has developed an anti-human IL-13 antibody that is effective in animal models of asthma [201,205] and IMA-638 (IgG1, kappa), a humanized antibody to human IL-13 from Wyeth Research is effective in animal models of asthma [202,206].

As for IL-4 (see above) a novel approach is represented by an IL-13 peptide-based vaccine for blocking IL-13 on a persistent basis. Vaccination significantly inhibits increase in inflammatory cell number and IL-13 and IL-5 levels in BAL. Serum total and ovalbumin-specific IgE are also significantly inhibited. Moreover, allergen-induced goblet cell hyperplasia, lung tissue inflammatory cell infiltration and AHR are significantly suppressed in vaccinated mice in an animal model of asthma [207].

IL-4 muteins indicate two types of IL-4 variants whose tyrosine at 124 is replaced with aspartate (Y124D) and arginine at 121 is replaced with aspartate (R121D/Y124D). IL-4 muteins act as antagonists for both IL4 and IL-13, because they are able to bind to IL-4R/IL13R, but do not transduce the signal. Bayer initially developed R121D/Y124D (pitrakinra, BY-16-9996, Aerovant) and now the compound is in phase IIa clinical trial for the treatment of asthma under license to Aerovance [203]. Promising results have been recently published on the efficacy of this compound in the prevention of late phase asthmatic responses on allergen challenge in asthmatics [203].

Novel “traps” composed of fusions between two distinct receptor components and a portion of the Fc region of the antibody molecule, result in the generation of blockers with markedly increased affinity over that offered by single component reagents, dual IL-4/IL-13 trap is in preclinical development for asthma ().

## IL-5

The Th2 cell cytokine, IL-5, plays an important role in eosinophil maturation, differentiation, recruitment, and survival. IL-5 knockout mice appeared to confirm a role in asthma models where eosinophilia and AHR is markedly suppressed. Humanised anti-IL-5 antibodies have been developed and a single i.v. infusion of one of these (mepolizumab) markedly reduces blood and sputum eosinophilia for several months. Unfortunately, there was no significant effect on the early or late response to allergen challenge, base-line AHR or FEV_1_[62]. A similar study in moderate/severe persistent asthma showed similar results on eosinophilia but with no improvements in symptoms or lung function [208]. In a subsequent study eosinophil numbers within the bronchial mucosa were only reduced by ∼50 by mepolizumab treatment but again no effect on lung function was noted [209]. These data have raised questions over the importance of eosinophils in asthma. In a controlled clinical trial, administration of mepolizumab, over a period of 6 months, to asthmatic patients markedly reduces peripheral blood eosinophils without altering the distribution of T-cell subsets and activation status (pattern of Th1 and Th2 cytokine production) of blood lymphocytes [210].

In recent studies RNA interference using a short hairpin RNA, has been able to block IL-5Rα expression, decrease bone marrow eosinophilopoiesis and blood and BAL eosinophilia in an animal model of asthma showing new potential blockers of IL-5 function [211,212]. These new compounds have still not been tested in human asthma.

## IL-6 antagonists and asthma

Interleukin-6 and related cytokines, interleukin-11, leukemia inhibitory factor, oncostatin M, ciliary neurotrophic factor, and cardiotrophin-1 are all pleiotropic and exhibit overlapping biological functions. Functional receptor complexes for the IL-6 family of cytokines share the signal transducing component glycoprotein 130 (gp130). Unlike cytokines sharing common β and common γ chains that mainly function in hematopoietic and lymphoid cell systems, the IL-6 family of cytokines function extensively outside these systems as well, owing to the ubiquitous expression of gp130 [213].

The IL-6 receptor complex (IL-6R) consists of either the membrane-bound IL-6 receptor (mIL-6R) or the soluble IL-6 receptor (sIL-6R) complexed with gp130. There are increased levels of sIL-6R in the airways of patients with allergic asthma as compared to those in controls. In addition, local blockade of the sIL-6R in a murine model of asthma led to suppression of Th2 cells in the lung [214]. By contrast, blockade of mIL-6R induced local expansion of Foxp3-positive CD4+CD25+ Tregs with increased immunosuppressive capacities. CD4+CD25+ but not CD4+CD25- lung T cells selectively expressed the IL-6Rα chain and showed IL-6-dependent STAT-3 phosphorylation. Finally, in an animal model of asthma CD4+CD25+ T cells isolated from anti-IL-6R antibody-treated mice exhibited marked immunosuppressive and antiinflammatory functions [214].

## IL-9 antagonists and asthma

Numerous *in vitro* and *in vivo* studies in both animals and patients with asthma have shown that IL-9 is an important inflammatory mediator in asthma. IL-9 is produced in the lung by a number of different cell types (Th2 lymphocytes, mast cells, eosinophils, bronchial epithelial cells), and has multiple effects on a wide range of inflammatory and structural cells within the lung, including bronchial epithelial and smooth muscle cells (release of CCL11). IL-9 may be involved in IL-4-triggered IgE production *in vitro*, mast cells and eosinophils recruitment and activation to the lung, bronchial mucus cell hyperplasia (and MUC4 induction), subepithelial deposition of collagen, and airway hyperresponsiveness [215]. Animal data indicate that IL-9 can promote asthma through IL-13-independent pathways via expansion of mast cells, eosinophils, and B cells, and through induction of IL-13 production by hemopoietic cells for mucus production and recruitment of eosinophils by lung epithelial cells [216]. IL-9 mRNA and protein is increased in the bronchial mucosa of atopic asthmatics, where it is expressed predominantly in lymphocytes [217,218]. In addition, BAL IL-9 levels are up-regulated in asthmatics following allergen challenge [219].

In animal models of asthma the overexpression of IL-9 causes BAL eosinophilia, peribronchial accumulation of collagen and increased BAL levels of CCL5 and CTGF [220].

However, in Th2 cytokine-deficient mice (IL-4, IL-5, IL-9, and IL-13; single to quadruple knockouts) IL-4 alone can activate all Th2 effector functions even in the combined absence of IL-5, IL-9, and IL-13 [221] and the Th2 pulmonary inflammation is unchanged in IL-9-deficient mice, despite a reduced number of lung mast cells and goblet cells [222]. Despite this, in an animal model of asthma the treatment with an anti-IL-9 antibody reduces airway inflammation and hyperresponsiveness [223,224] suggesting that blockade of IL-9 may be a new therapeutic strategy for bronchial asthma [215]. Interestingly, after allergen exposure an anti-IL-9 antibody significantly reduces bone marrow eosinophilia in an animal model primarily by decreasing newly produced and mature eosinophils. Anti-IL-9 treatment also reduces blood neutrophil counts, but does not affect BAL neutrophils [225].

## IL-10 modulation and asthma

New "counterregulatory" models of asthma pathogenesis suggest that dysfunction of IL-10–related regulatory mechanisms might underlie the development of asthma.

IL-10 is produced by several cell types, including monocytes, macrophages, T lymphocytes, dendritic cells and mast cells. IL-10 is a unique cytokine with a wide spectrum of anti-inflammatory effects. It inhibits the secretion of TNFα and IL-8 from macrophages and tips the balance in favour of antiproteases by increasing the expression of endogenous tissue inhibitors of MMPs (TIMPS). Some of the actions of IL-10 can be explained by an inhibitory effect on NF-κB, but this does not account for all effects, as IL-10 is very effective at inhibiting IL-5 transcription, which is independent of NF-κB. In mice many effects of IL-10 appear to be mediated by an inhibitory effect on PDE-4, but this does not appear to be the case in human cells [191].

However in animal models, IL-10, although inhibiting lipopolysaccharide-induced airway inflammation, also causes airway mucus metaplasia, inflammation, and fibrosis. These responses are mediated by multiple mechanisms with airway mucus metaplasia being dependent on the IL-13/IL-4Rα/STAT-6 activation pathway whereas the inflammation and fibrosis is independent of this pathway [226].

IL-10 concentrations are reduced in induced sputum from patients with asthma or COPD, indicating that this might be a mechanism for increasing lung inflammation in these diseases. In addition, IL-10 production is decreased in peripheral blood mononuclear cells of patients with mild asthma and is further attenuated in severe persistent asthma compared to mild asthma [227,228]. Patients with severe persistent asthma have increased frequency of a haplotype associated with low production of IL-10 by the alveolar macrophages [229]. Furthermore, a defect in glucocorticoid-induced IL-10 production has also been described in blood T lymphocytes from patients with glucocorticoid-resistant asthma [228].

The potent immunosuppressive and anti-inflammatory action of IL-10 has suggested that it may be useful therapeutically in the treatment of asthma. Recombinant human IL-10 has already been licensed for Crohn's disease and psoriasis by daily subcutaneous injection over 4 weeks and it is reasonably well tolerated causing only a reversible dose-dependent anemia and thrombocytopenia. Another possibility for therapy in the future is the development of other agonists for the IL-10 receptor, or drugs that activate the unique, but so far unidentified, signal transduction pathways activated by this cytokine [191].

Recent data also suggests that vitamin D3 in conjunction with a glucocorticoid may restore the reduced expression of Il-10 seen in T-cells from patients with severe asthma [228].

## IL-12 modulation and asthma

IL-12 is essential for the development of Th1 immune response, leading to their production of IFN-γ. In addition to priming CD4+ T cells for high IFN-γ production, IL-12 also contributes to their proliferation once they have differentiated into Th1 cells. IL-12 is also capable of inhibiting the Th2-driven allergen-induced airway changes in mice and was therefore considered a new potential drug for the treatment of asthma. In man, IL-12 production is decreased in PBMCs, alveolar macrophages and bronchial biopsies of patients with mild asthma and IL-12 synthesis is further attenuated in PBMCs from severe persistent asthma compared to mild asthma [227]. Inhalation of IL-12 has been shown to inhibit allergic inflammation in murine models while decreasing adverse effects seen with systemic administration of this cytokine and adenoviral IL-12 gene transduction may be effective in inducing IL-12 expression in the airways [230]. However, an initial study of inhaled IL-12 in humans with asthma was terminated because of adverse effects, including one death. Furthermore, the use of systemically administered IL-12 in patients with asthma has been limited due to cytokine toxicity and lack of clinical efficacy despite a significant reduction in the number of blood and sputum eosinophils [231]. Another treatment option that has the potential of inducing a Th1 cytokine response is the use of IL-12 linked to polyethylene glycol moieties. This mode of administration is likely to enhance cytokine delivery to the target organ, while decreasing its toxicity. Also intranasal delivery of IL-12 may provide another approach for the treatment of asthma [232].

## IL-15

The IL-15 gene is located on chromosome 4q27, approximately distal to the IL2 gene and may be associated with an increased susceptibility to asthma [233,234].

IL-15 shares many biologic activities with IL-2. Both cytokines bind a specific α subunit, and they share the same β and γ common receptor subunits for signal transduction. IL-15, in the presence or absence of TNF-α, reduces spontaneous apoptosis in human eosinophils. The number of cells expressing IL-15 is significantly increased in the bronchial mucosa from patients with Th1-mediated chronic inflammatory diseases of the lung such as sarcoidosis, tuberculosis, and COPD compared with asthmatic patients and normal subjects [235]. The expression of IL-15 is also increased in the bronchial mucosa of asthmatic patients compared to normal subjects.

In an animal model of asthma overexpression of IL-15 suppresses Th2-mediated-allergic airway response via induction of CD8+ T cell-mediated Tc1 response [236]. However in another animal model of asthma blocking IL-15 prevents the induction of allergen-specific T cells and allergic airway inflammation [237]. Natural killer (NK) cells are divided into NK1 and NK2 subsets and the ratio of IL-4 + CD56 + NK2 cells in PBMCs of asthmatic patients is higher than in healthy individuals [238]. STAT6 is also constitutively activated in NK2 clones from asthmatic patients possibly as a result of IL-15 stimulating their proliferation [238]. There are no clinical studies on the effect of IL-15 pathway modulation in asthmatic patients. Interestingly, a two weeks treatment with the inhaled glucocorticoid fluticasone decreased the numbers of IL-15+ cells in the bronchial mucosa of stable asthmatics [239].

## IL-18 modulation and asthma

IL-18, originally identified as an IFN-γ-inducing factor, is a unique cytokine that enhances innate immunity and both Th1- and Th2-driven immune responses. IL-18 is able to induce IFN-γ, GM-CSF, TNFα and IL-1, to activate killing by lymphocytes, and to up-regulate the expression of certain chemokine receptors. In contrast, IL-18 induces naive T cells to develop into Th2 cells. IL-18 also induces IL-4 and/or IL-13 production by NK cells, mast cells and basophils [240].

The same dualism is present *in vivo* after administration of IL-18 in animal models of asthma. Vaccination with allergen-IL-18 fusion DNA protects against, and reverses established, airway hyperresponsiveness in an animal model of asthma [241]. On the other hand, in other animal models, administration of IL-18 enhances antigen-induced increase in serum IgE and Th2 cytokines and airway eosinophilia in part by increasing antigen-induced TNFα production [242,243]. This suggests that IL-18 may contribute to the development and exacerbation of Th2-mediated airway inflammation in asthma [244,245].

The serum levels of IL-18 are higher in asthmatic patients [246] and increase further during exacerbations (and decrease during the stable phase) compared with normal subjects [247]. Decreased levels of IL-18 in sputum and BAL from asthmatic patients compared to normal controls have also been reported [248,249]. There are no clinical studies on the effect of the administration of human recombinant IL-18 and/or IL-18 antagonists to asthmatic patients.

## Class II family of cytokine receptors

Class II family of cytokine receptors (CRF2) now includes 12 proteins: a new human Type I IFN, IFN-κ; molecules related to IL-10 (IL-19, IL-20, IL-22, IL-24, IL-26); and the IFN-λ proteins IFN-λ1 (IL-29), IFN-λ2 (IL-28A), and IFN-λ3 (IL-28B), which have antiviral and cell stimulatory activities reminiscent of type I IFNs, but act through a distinct receptor and are designated as type III IFN by the nomenclature committee of the International Society of Interferon and Cytokine Research [250,251]. In response to ligand binding, the CRF2 proteins form heterodimers, leading to cytokine-specific cellular responses and these diverse physiological functions are just beginning to be explored. The ligand-binding chains for IL-22, IL-26, and IFN-λ are distinct from that used by IL-10; however, all of these cytokines use a common second chain, IL-10 receptor-2 (IL-10R2; CRF2-4), to assemble their active receptor complexes. Thus, IL-10R2 is a shared component in at least four distinct class II cytokine-receptor complexes. IL-10 binds to IL-10R1; IL-22 binds to IL-22R1; IL-26 binds to IL-20R1; and IFN-λ binds to IFN-λR1 (also known as IL-28R) [253-256]. The binding of these ligands to their respective R1 chains induces a conformational change that enables IL-10R2 to interact with the newly formed ligand-receptor complexes. This in turn activates a signal-transduction cascade that results in rapid activation of several transcription factors, particularly STAT3 [252] and to a lesser degree, STAT1 [253-256].

## IL-19 modulation and asthma

IL-19 belongs to the IL-10 family, which includes IL-10, IL-19, IL-20, IL-22, IL-24 [(melanoma differentiation-associated gene-7 (MDA-7)], and IL-26 (AK155). The IL-19, IL-20, and IL-24 genes are on chromosome 1q31–32, a region that also contains the IL-10 gene. The two other IL-10-related cytokines, IL-22 and IL-26 genes are on chromosome 12q15, near the IFN-γ gene [252]. IL-19 and IL-24 bind to the type I IL-20R complex which is a heterodimer of two previously described orphan class II cytokine receptor subunits: IL-20R1 [IL-20Rα or corticotropin-releasing factor (CRF) 2–8], and IL-20R2 [IL-20Rβ (DIRS1)] [252,257]. In addition, IL-20 and IL-24 but not IL-19, bind to type II IL-20R complex, composed of IL-22R1 and IL-20R2 [252]. In all cases, binding of the ligands results in STAT3 phosphorylation [252].

The IL-19 gene is up-regulated in monocytes by LPS and GM-CSF [258] and, in turn, IL-19 induces the production of IL-6, TNF-α and oxidants in these cells [259]. IL-19 can also induces apoptosis in monocytes [259]. IL-19 also induces the Th2 cytokines IL-4, IL-5, IL-10, and IL-13 production by activated T cells [257,260]. *In vitro*, A2B adenosine receptors induce IL-19 from bronchial epithelial cells, resulting in TNF-α release [261].

The serum level of IL-19 in patients with stable asthma is increase compared with healthy controls [260]. In an animal model of asthma there is increased IL-19 expression and transfer of the IL-19 gene into healthy mice up-regulated IL-4 and IL-5, but not IL-13, however, IL-19 up-regulated IL-13 in “asthmatic” mice [260]. The role of IL-19 blockers in asthma needs to be explored.

## IL-21 modulation and asthma

The interleukin-2 family of cytokines includes IL-2, IL-4, IL-7, IL-9, IL-13, IL-15 and IL-21. The IL-21 gene is located on human chromosome 4q26-27, near the IL-2 gene. In humans IL-21 is produced almost exclusively by CD4^+^ Th1 and Th2 cells. There is very little expression of IL-21 in activated CD8^+^ cells [262]. The IL-21 receptor complex is a heterodimer containing the IL-21R and the common cytokine receptor γ chain (γc) of the IL-2, IL-4, IL-7, IL-9, and IL-15 receptors [263]. IL-21 binding stimulates activation of Janus kinase (JAK)1/JAK3 and then preferentially activates STAT-1 and STAT-3 [263]. In addition, IL-21 enhances STAT4 binding to the IFN-γ promoter.

IL-21 modulates the proliferation and differentiation of T cells towards a Th1 phenotype and also stimulates B cells, NK cells, and dendritic cells [262,263]. In addition, IL-21 also stimulates IgG1 production and decreases IgE production [264]. Thus, IL-21 may be a critical cytokine maintaining low IgE levels under physiological and pathological conditions and importantly in support of this IL-21 knockout animals have an increased level of serum IgE and IgE producing B cell expansion [265]. Interestingly, IL-21 knockout and IL-21R knockout animals are healthy and fail to acquire spontaneous inflammatory diseases [264,266].

IL-21 is also a potent stimulator of cell-mediated immunity (effector CD8^+^ T and NK cells) and it has a potent anti-tumour activity in many animal models [262,267] and ZymoGenetics (.) is developing IL-21 for the treatment of cancer.

IL-21 administration in an animal model of asthma reduces titres of antigen-specific IgE and IgG1 antibodies, as well as airway hyperresponsiveness and lung eosinophil recruitment [262]. Thus, IL-21 signalling modulation may be useful for the treatment of asthma [268].

## IL-22 modulation and asthma

The IL-22 gene (and also the IL-26 gene) is located on human chromosome 12q. The IL-22 heterodimeric receptor is composed of the IL-22R1 (CRF2–9/IL-22R subunit )and the IL-10R2 to generate the IL-22 receptor complex, or with IL-20R2 to yield another receptor complex for IL-20 and IL-24 [269]. In addition to its cellular receptor, IL-22 binds to a secreted member of the class II cytokine receptor family, which is called IL-22BP, a soluble receptor which is a naturally occurring IL-22 antagonist.

There are several lines of evidence connecting IL-22 to asthma. Interestingly, *in vitro* long-term (12 days) exposure of human T cells to IL-19, IL-20 and IL-22 down-regulated IFN-gamma but up-regulated IL-4 and IL-13 and supported the polarization of naive T cells to Th2-like cells. In contrast, neutralization of endogenous IL-22 activity by IL-22-binding protein decreased IL-4, IL-13 and IFN-gamma synthesis [270].

IL-22 is induced by IL-9, a Th2 cytokine potentially involved in asthma (see above) and by LPS in animal models of asthma [269]. IL-22 induces *in vitro* and *in vivo* expression of several acute phase proteins, β-defensins, pancreatitis-associated protein (PAP1) and osteopontin [269]. Some of these proteins are involved in inflammatory and innate immune responses. Inasmuch as IL-22 is implicated in inflammation, the expression of IL-22BP should decrease local inflammation. In this light, it is of interest that IL-22BP expression was detected by *in situ* hybridization in the mononuclear cells of inflammatory infiltration sites, plasma cells, and a subset of epithelial cells in several tissues including lung [271]. Thus, IL-22 signalling modulation may be useful for the treatment of asthma.

## IL-23 modulation and asthma

IL-23 and IL-27 are heterodimeric cytokines functionally and structurally related to IL-12. Along with two other cytokines, CLC/soluble CNTFR and CLC/CLF-1, IL-12, IL-23, and IL-27 compose the IL-12 cytokine family [272]. IL-23 is a dimer composed of the IL-12p40 subunit and the IL-12p35-related molecule p19 and is produced mainly by macrophages and dendritic cells. The heterodimerized IL-23 receptor is composed of a shared IL-12 receptor beta 1 (IL-12Rβ1; p40) and an IL-12Rβ2-related molecule called IL-23R (p19), expressed in natural killer and CD3+ CD4+ T cells. At least six spliced isoforms of IL-23R (IL-23R1 to 6) can be generated through alternative splicing (most often through deletion of exon 7 and/or exon 10) [272]. IL-23 is important for the proliferation of memory type Th1 cells [273] and promotes Th17 differentiation [274,275]. IL-23 is likely, therefore, to be important for the recruitment and activation of a range of inflammatory cells that is required for the induction of chronic inflammation.

The potential role of IL-23 modulation in the pathogenesis of asthma has still to be explored.

## IL-25 modulation and asthma

A novel IL-17 family cytokine IL-25 (IL-17E) is a product of activated Th2 cells and mast cells (via an IgE-dependent mechanism). Interestingly, when systemically administered to mice, IL-25 induces IL-4, IL-5 and IL-13 production from undefined non-T/non-B cells and then induces Th2-type immune responses such as blood eosinophilia and increased serum immunoglobulin E levels [276]. In addition, IL-25 mRNA is expressed in the lung in an animal model of asthma and neutralization of the produced IL-25 by soluble IL-25 receptor decreases antigen-induced eosinophil and CD4+ T cell recruitment into the airways. In contrast, overexpression of IL-25 in the lung significantly enhances antigen-induced Th2 cytokine production and eosinophil recruitment into the airways [276]. Thus, IL-25 may play an important role in enhancing allergic airway inflammation and is therefore a possible target for inhibition in the treatment of asthma.

## IL-27 modulation and asthma

IL-27 is composed of the Epstein-Barr virus (EBV)-induced gene 3 (EBI3) protein (this subunit has sequence homology with IL-12p40) plus the IL-12p35-related protein p28 [277]. A polymorphism of the IL-27p28 gene is associated with susceptibility to asthma [278]. Most of the IL-27 is produced by macrophages and dendritic cells. The functional IL-27 receptor complex is a heterodimeric receptor composed of either the IL-27R *α* chain [WSX-1 (after the WSXWS motif, a characteristic feature of cytokine receptors in its extracytoplasmic portion) or the T-cell cytokine receptor (TCCR)], in combination with gp130 [279]. The IL-27R *α* chain is highly expressed on CD4^+^ T lymphocytes and NK cells and ligand binding leads to STAT1 and STAT3 activation [280]. As has been observed with other members of the IL-6/IL-12 family, IL-27 has a double identity as an initiator and as an attenuator of immune responses and inflammation [281]. As such, IL-27 can function as a proinflammatory cytokine because it synergises with IL-12 to induce IFN-γ production from NK cells and promotes Th1 differentiation through the induction of T-bet and the activation of STAT4.

Whilst IL-12 is the most potent inducer of Th1 differentiation and IFN-*γ* production acting on effector Th1 cells, chronologically differential roles and differential usage of IL-12, IL-23, and IL-27 have been proposed. First, IL-27 commits naïve CD4^+^ T cells to differentiate into Th1 cells by inducing IL-12R*β*2, then IL-12 acts on committed effector Th1 cells for IFN-*γ* production, followed by IL-23 mediating the proliferation of memory Th1 cells [281]. IL-27 also has anti-inflammatory properties. IL-27 inhibits (through a STAT1- and STAT3-dependent pathway) the development of Th17 cells [282,283]. IL-23 also inhibits the development of the iTreg cells [282]. It is believed that the activation status of the cells may be the key determinant for the differing effects of IL-27. IL-27 acts on naïve T cells for IFN-γ production while the same cytokine suppresses cytokine production by affecting fully activated cells [281].* In vitro* IL-27 also inhibits Th2 cell differentiation through decreased expression of GATA-3 and subsequent reduction in IL-4 production [284,285].

In animal models of allergic asthma knockout of IL-27R*α* chain results in a marked increased of airway hyperresponsiveness, BAL eosinophilia, goblet cell hyperplasia and serum IgE levels [286]. Production of the Th2 cytokines IL-4 and IL-13 is also augmented in the BAL, but, surprisingly, IFN-γ production is also enhanced, albeit to a low level [286]. The role of IL-27 modulation in human asthma is still unknown.

## IL-31 modulation and asthma

IL-31 gene is located on chromosome 12q24.31 and is predominantly expressed by activated CD4+ T cells, particularly of the Th2 phenotype [287]. The IL-31 receptor is an herodimer complex formed by IL-31Rα (gp130-like protein), a member of the IL-6R group that does not engage gp130, and the oncostatin M receptor β (OSMRβ) that is shared with oncostatin M, to form a signaling receptor complex [287,288]. This receptor is constitutively expressed in lung epithelium and is induced in activated monocytes [287]. Lung epithelial cells express high levels of IL-31Rα, OSMRβ and gp130 but despite this IL-31 can utilize a distinct set of intracellular signaling pathways compared to oncostatin M which acts through the OSMRβ/gp130 complex to produce different functional effects [289]. This, in the A549 cells, initiates IL-31 signalling that differs from other IL-6 cytokines by the particularly strong recruitment of the STAT3, ERK, JNK, and Akt pathways. IL-31 is highly effective in suppressing proliferation by altering expression of cell cycle proteins, including up-regulation of p27 and down-regulation of cyclin B1, CDC2, CDK6, MCM4, and retinoblastoma. A single STAT3 recruitment site (Tyr-721) in the cytoplasmic domain of IL-31Ralpha exerts a dominant function in the entire receptor complex and is critical [289]. IL-31 also appears to be an important regulator of Th2 responses. IL-31 transgenic mice, shows pruritis and dermatitis (with an inflammatory cell infiltrate rich of T lymphocytes similar to that observed in atopic dermatitis) with lesions in the skin and dorsal root ganglia [290-292]. Importantly, the expression of IL-31 is increased in the skin of patients with atopic dermatitis and correlates with IL-4 and IL-13 expression [293]. In an animal model of asthma antigen challenge increases the expression of IL-31 in lung epithelium and BAL cells [287]. However, more recent data suggest a novel role for IL-31R signaling in specifically limiting type 2 inflammation in the lung [294]. *In vitro*, macrophages generated from IL-31Rα knockout animal promoted enhanced allergen-specific CD4+ T cell proliferation and these cells exhibited enhanced proliferation and expression of Th2 cytokines, identifying a T cell- and macrophage-intrinsic regulatory function for IL-31R signaling [294]. In contrast, the generation of CD4+ T cell-mediated Th1 responses is normal in IL-31R knockout, suggesting that the regulatory role of IL-31R signaling is limited to Th2 responses [294]. These data suggest that modulation of IL-31 expression may be in the future another target for new drugs for asthma.

## IL-33 modulation and asthma

The cytokines of the IL-1 family; IL-1α/β, IL-1Ra, and IL-18 have been matched to their respective receptor complexes. Recently, the ligand for the most prominent orphan IL-1 receptor, T1/ST2 [295] was found to be IL-33 [296]. Three distinct types of ST2 (also known as T1, Fit-1, and DER4) gene products have been cloned; a soluble secreted form (ST2), a transmembrane receptor form (ST2L), and a variant form (ST2V). ST2L is preferentially expressed on Th2 cells [297] and stimulates Th2 cytokine production in the lung and functions as an important effector molecule of Th2 (but not Th1) responses [298,299]. Studies in ST2L-deficient mice have produced conflicting results [300-302] but suggest that while ST2L can be bypassed by alternative mechanisms following multiple antigenic challenges, it probably plays a key role in the early events involved in the generation of Th2 responses [303]. Interestingly, expression of GATA-3, CCR3, -4, -8, and ST2L, and the generation of blood eosinophilia, is intact in mice doubly deficient in both IL-4 and IL-13 [304]. ST2L has also been described as a negative regulator of Toll-like receptor4 -IL-1 receptor signaling maintaining endotoxin tolerance [305].

Additionally, ST2L is able to activate the transcription factor AP-1; increase phosphorylation of c-Jun, and activate the MAP kinases c-Jun N-terminal kinase (JNK), p42/p44 and p38.

Anti-T1/ST2 also induces the selective expression of IL-4 but not IFN-γ in naive T cells. Importantly, this effect is blocked by prior treatment with the JNK inhibitor SP600125 confirming that JNK as a key effector in T1/ST2 signaling [306]. Furthermore, IL-33 activates NF-κB and MAP kinases, and drives production of Th2-associated cytokines from *in vitro* polarized Th2 cells [295].

In animals, IL-33 treatment induces the expression of IL-4, IL-5 and IL-13 in the lung [295] and intraperitoneal injection of IL-33 induces spleen eosinophilia [295].

IL-33 is not only able to elucidate effects through binding to ST2L but, in a manner analogous to that of IL-1α and HMGB1, can act directly in the nucleus colocalizing with mitotic chromatin through an evolutionarily conserved homeodomain-like helix-turn-helix motif within the IL-33 N-terminal domain and possesses transcriptional repressor properties [296].

Soluble ST2 protein levels are elevated in the sera of patients with asthmatic exacerbation, and correlate well with the severity of asthma exacerbation [307]. Some studies suggest that soluble ST2 might have an autoregulatory role in animal models of asthma [308]. However, if signalling via ST2L is in fact only important during the induction of a Th2 response, then production of soluble ST2 in response to antigenic challenge when allergic sensitization has already been established seems unlikely to achieve much in terms of autoregulation. Consistent with this, in transgenic mice with high serum levels of a soluble ST2-Ig recombinant protein, which would be expected to bind to and block the putative ligand, there is minimal reduction in Th2-dependent pulmonary inflammation [301,303].

Animal models of allergic asthma have demonstrated an increased accumulation of lung CD4+/ST2+ Th2 cells [309] and ST2 knockout animals have a significant decrease of the production of Th2 cytokines [302]. Monoclonal anti-T1/ST2 treatment reduces lung inflammation and the severity of illness in mice infected with RSV, a model of Th2-mediated immunopathology of the lung [310]. These data suggest that modulation of IL-33 expression and/or action is a potential target for new drugs for asthma.

## Other modulators of the Th1/Th2 balance in asthma

### Surfactant proteins and asthma

Treating animal models of asthma with surfactant proteins can suppress IgE levels, eosinophilia, pulmonary cellular infiltration and cause a marked shift from a Th2 to a Th1 cytokine profile [311-313].

However, a natural porcine surfactant preparation (Curosurf) given before segmental allergen challenge to mild asthmatics unexpectedly, augmented the eosinophilic inflammation 24 hours after allergen challenge. A direct chemotactic effect of Curosurf was excluded. However, levels of eotaxin and IL-5 were increased in BAL after Curosurf treatment, whereas IFN-γ-levels and numbers of IFN-γ+ T cells were decreased. Curosurf had no influence on spreading and retention of allergen determined by allergen uptake in mice [314].

### Suplatast

Suplatast tosilate is a novel oral anti-asthma compound that, *in vitro*, selectively inhibits IL-4 and IL-5 production from allergen-stimulated human Th2 lymphocytes, but not IFN-γ production from human Th1 lymphocytes. Suplatast may also prevent allergen-induced goblet-cell metaplasia and attenuates inflammatory mediators-induced eosinophil chemotaxis and eosinophil adhesion to endothelial cells [8]. Suplatast in a small placebo-controlled clinical trial has been effective as inhaled beclomethasone in improving FEV_1_ and mean morning PEF. In patients with moderate asthma (not treated with long-acting bronchodilators), suplatast can significantly decrease the use of the inhaled glucocorticoid.

In other small clinical trials, suplatast decreased blood and sputum eosinophils, blood and sputum eosinophil cationic protein levels, sputum mast cell tryptase levels, exhaled nitric oxide and airway responsiveness in patients with mild-moderate asthma or cough-variant asthma without causing significant side-effects [8].

### Immunomodulatory effects of helminth products and asthma

Epidemiological studies suggest that helminthic hookworms infections may protect subjects from developing asthma [315]. In an animal model of asthma the application of excreted/secreted products (NES) of the helminth Nippostrongylus brasiliensis together with ovalbumin/alum during the sensitization period totally inhibited the development of eosinophilia and goblet cell metaplasia in the airways and also strongly reduced the development of airway hyperresponsiveness [316]. Allergen-specific IgG1 and IgE serum levels are also strongly reduced. These findings correlated with decreased levels of IL-4 and IL-5 in the airways in NES-treated animals [316]. The suppressive effects on the development of allergic responses were independent of the presence of Toll-like receptors 2 and 4, IFN-γ, and IL-10. Paradoxically, strong helminth NES-specific Th2 responses are induced in parallel with the inhibition of asthma-like responses [316,317].

Th2 responses induced by allergens or helminths share many common features. However, allergen-specific IgE can almost always be detected in atopic patients, whereas helminth-specific IgE is often not detectable and anaphylaxis often occurs in atopy but not with helminth infections [318]. This may be due to T regulatory responses induced by the helminths or the lack of helminth-specific IgE. Alternatively non-specific IgE induced by the helminths may protect from mast cell or basophil degranulation by saturating IgE binding sites. Both of these mechanisms have been implicated to be involved in helminth-induced protection from allergic responses [318]. However a recent study has shown that N. brasiliensis antigen (Nb-Ag1) specific IgE could only be detected for a short period of time during infection, and that these levels are sufficient to prime mast cells thereby leading to active cutaneous anaphylaxis after the application of Nb-Ag1. Taken together, at least for the model helminth *N. brasiliensis*, the IgE blocking hypothesis can be discarded [319]. However, novel antigens binding helminth-specific IgE may be identified for other pathogenic helminths infecting humans. Identifying these antigens may aid in IgE/mast cell-dependent vaccine development for asthma [318].

Epidemiological studies suggest that a hookworm infection producing 50 eggs/gram of faeces may protect against asthma [320]. A pilot dose-ranging study of experimental human infection with Necator americanus larvae has been performed to identify the dose of hookworm larvae necessary to achieve 50 eggs/gram of faeces for therapeutic trials in asthma [321]. A controlled clinical trial with Necator americanus larvae in asthmatics is now underway [322] ().

Despite potential safety concerns (cutaneous larva migrans and/or Loffler's syndrome or other pulmonary complications [323,324] about this approach, the experimental infection of a small number of human subjects has been surprisingly safe [321,325].

## Conclusions

The current asthma therapies are not cures and symptoms return soon after treatment is stopped even after long term therapy. Although glucocorticoids are highly effective in controlling the inflammatory process in asthma, they appear to have little effect on the lower airway remodelling processes that appear to play a role in the pathophysiology of asthma at currently prescribed doses. The development of novel drugs may allow resolution of these changes. In addition, severe glucocorticoid-dependent and resistant asthma presents a great clinical burden and reducing the side-effects of glucocorticoids using novel steroid-sparing agents is needed. Furthermore, the mechanisms involved in the persistence of inflammation are poorly understood and the reasons why some patients have severe life threatening asthma and others have very mild disease are still unknown. Considering the apparently central role of T lymphocytes in the pathogenesis of asthma, drugs targeting disease-inducing Th2 cells are promising therapeutic strategies [326]. However, although animal models of asthma suggest that this is feasible, the translation of these types of studies for the treatment of human asthma remains poor due to the limitations of the models currently used. Since we do not yet understand the underlying causes of asthma it is unlikely that therapy will lead to a cure.

The myriad of new compounds that are in development directed to modulate Th2 cells recruitment and/or activation will clarify in the near future the relative importance of these cells and their mediators in the complex interactions with the other pro-inflammatory/anti-inflammatory cells and mediators responsible of the different asthmatic phenotypes. Hopefully, it will soon be possible to identify and manipulate the molecular switches that result in asthmatic inflammation. This may lead to the treatment of susceptible individuals at birth or in the early years and thus prevent the disease from becoming established.

## Competing interests

The authors declare that they have no competing interests.

## Authors' contributions

All the authors have contributed equally to this work.
